# Health support for people on the move: a qualitative exploration of civil society organizations’ roles and obstacles along the Balkan route

**DOI:** 10.1186/s12939-026-03048-x

**Published:** 2026-09-24

**Authors:** Fabian Link, Zeliha Öcek, Apostolos Veizis, Michaela Coenen

**Affiliations:** 1 Institute for Medical Information Processing, Biometry, and Epidemiology – IBE, Chair for Public Health and Health Services Research, LMU Medizin, LMU Munich, Munich, Germany; 2 Pettenkofer School of Public Health, Munich, Germany; 3 INTERSOS HELLAS, Athens, Greece

**Keywords:** Migrants, Refugees, People on the move, Civil society organizations, Health care delivery, Access to health care, Qualitative research

## Abstract

**Background:**

People on the move (POTM) are individuals in ongoing migration processes, often facing significant health risks and structural barriers to equitable access to essential services despite international frameworks recognizing healthcare rights. Civil society organizations (CSOs) play a key role in addressing these gaps. This study examines CSOs’ health support for POTM from the perspective of CSO workers, using the Balkan Route as a case study.

**Methods:**

This qualitative study included CSO staff providing health-related support to POTM along the Balkan Route. Participants were recruited through purposive and snowball sampling across 11 countries. Data were collected between March and May 2024 through semi-structured, in-depth interviews addressing healthcare access, service gaps, CSO roles, challenges, and institutional relations. Interviews were analyzed using qualitative content analysis.

**Results:**

Fifteen CSO workers from 11 countries participated, representing local, national, and international CSOs. Analysis identified four main categories: (1) Structural and Sociopolitical Context (2), POTM (3), National Healthcare Systems (4), CSOs. Participants described fragmented migration governance, limited health system capacities, discrimination, and legal barriers as key determinants of healthcare access. POTM experienced cumulative health risks and multiple structural, administrative, and communication barriers. Although states were viewed as primarily responsible for healthcare provision, participants consistently reported that CSOs filled critical gaps by delivering health-related services, facilitating access through mediation, and advocating for more equitable healthcare. However, these roles were constrained by unstable funding, workforce shortages, political pressures, and ethical tensions regarding the substitution of state responsibilities. Trust and collaboration between CSOs, healthcare providers, public authorities, local communities, and POTM emerged as essential for effective healthcare support.

**Conclusions:**

CSOs are central to healthcare in supporting healthcare access for POTM, particularly through their mediating role between migrants, healthcare systems, and public authorities. Strengthening trust-based collaboration while preserving CSO independence and maintaining state accountability may contribute to more equitable healthcare access in migration contexts.

**Supplementary Information:**

The online version contains supplementary material available at https://doi.org/10.1186/s12939-026-03048-x.

## Background

Ongoing conflicts, political instability, and economic hardship across the Middle East, Southeast Asia, and Africa continue to drive migration towards Europe, often via irregular routes [1, 2]. In 2025, around 178,000 irregular border crossings were recorded at the European Union’s external borders, a 26% decrease from 2024, although the Central and Eastern Mediterranean and Western Balkans routes remain key pathways [3]. These flows are commonly described as mixed migration, involving people travelling along similar routes for different reasons, including fleeing conflict or seeking better socio-economic conditions [4]. The term People on the Move (POTM) reflects this heterogeneous population [5].

European migration and asylum governance has increasingly been shaped by the securitisation of migration, with policies prioritising border control, the management of irregular migration, and the externalisation of migration governance over protection-oriented approaches. This shift has fundamentally reshaped the legal and social conditions under which POTM travel, remain in transit, and access essential services, including healthcare. By restricting access to protection and legal pathways while reinforcing border controls, these policies have left many POTM in prolonged situations of legal uncertainty, limiting their access to rights and essential services [6]. Following the 2016 EU-Türkiye Statement and the closure of the Balkan route, short-term transit increasingly gave way to prolonged periods of waiting in transit countries [7].

As POTM spent longer periods in transit countries, their healthcare needs shifted from predominantly acute conditions to increasingly complex and long-term care needs [7, 8]. Cumulative exposure to physical and sexual violence, legal insecurity, inadequate shelter, interrupted access to healthcare, and poor living conditions contribute to communicable and non-communicable diseases, mental health problems, and sexual and reproductive health needs throughout the migration journey [5, 9–13]. Children are particularly vulnerable to malnutrition, injuries, disrupted routine vaccination, developmental risks, and exploitation [14], while women and girls face additional risks related to sexual and gender-based violence and exploitation [11]. Despite these diverse and evolving healthcare needs, access to healthcare for POTM remains limited across Europe, even in countries committed to universal health coverage. Although international and regional frameworks, including Article 25 of the Universal Declaration of Human Rights and the European Union’s Reception Conditions Directive, recognise the right to healthcare [15–22], access remains constrained by legal, administrative, linguistic, and organisational barriers, variations in healthcare entitlements, limited continuity of care, and insufficient coordination across health and social care services [8, 11]. Consequently, substantial gaps often persist between formal healthcare entitlements and actual access to healthcare, even where legal rights exist [23].

These persistent gaps between healthcare entitlements and actual access have made civil society organisations (CSOs) increasingly important actors in responding to the healthcare needs of POTM [23, 24]. Although no universally accepted definition exists, the United Nations broadly defines CSOs as citizen-led organisations operating across different areas of society [25, 26]. In the migration context, their close engagement with POTM, flexibility in adapting to changing contexts, and ability to reach populations who may remain underserved by formal institutions position them to complement formal efforts to ensure access to healthcare by supporting navigation across health and social care systems and advocating for more equitable access to care [23, 24]. Understanding the roles of CSOs and the challenges they encounter in responding to the healthcare needs of POTM is therefore essential for identifying health system gaps and informing future policies and service responses.

### Balkan route and the role of civil society organizations for people on the move

Against this broader European context, the Balkan Route (BR) provides a particularly relevant setting for examining how CSOs respond to structural barriers affecting healthcare for POTM. This corridor includes Albania, Bosnia and Herzegovina, Kosovo, Montenegro, North Macedonia, and Serbia [27, 28], but remains fluid, extending to countries such as Bulgaria and Croatia, as well as Türkiye and Greece, and destination countries including Austria and Germany in response to policy and geopolitical pressures [28–30]. Emerging in the early 2010s, the BR became a major European pathway, peaking in 2015 with around 200,000 people. Following the route’s formal closure in 2016, crossings declined but have fluctuated in recent years [28–30]. Studies from the BR document a broad range of physical, infectious, and mental health conditions among POTM, placing considerable demands on healthcare systems along the route. However, fragmented healthcare systems, differences in national policies, and legislative, financial, administrative, and practical barriers continue to challenge the continuity, coordination, and accessibility of healthcare across the route [7, 8, 10, 31–34].

Nearly a decade after the peak of the so-called “refugee crisis” and years after Kentikelenis et al. [24] highlighted the lack of systematic understanding of CSO involvement, the literature on healthcare along the BR has expanded. Existing studies have examined healthcare access [8, 35], camp settings [36], ethical challenges [23], and broader health provision [24]. Some studies report significant operational challenges, including limited resources, interpreter shortages, and fragmented coordination among CSOs and with governmental actors, contributing to parallel structures and inefficient use of resources [7, 8, 37, 38]. In some cases, fear of deportation leads POTM to avoid seeking care, hindering CSOs’ ability to establish contact and provide support [8].

Existing studies largely focus on experiences of POTM, with limited attention to CSO staff, their specific challenges, and their role in supporting healthcare. Studies examining multiple stakeholders [7, 8] have also tended to neglect national or grassroots CSOs [24, 38] or focus on healthcare professionals and service delivery rather than CSOs themselves [39–41]. To address these gaps, this study aimed to explore the roles of CSOs in supporting the health of POTM along the BR and the challenges they encounter. Specifically, the study was guided by the following research questions: [1] What roles do CSOs play in supporting the health of POTM along the BR [2]? What capacities and constraints shape CSOs’ ability to fulfil these roles [3]? How do structural and sociopolitical conditions, as well as relationships with POTM, local communities, healthcare systems, and public authorities, shape the roles of CSOs?

## Methods

### Design, study setting and participants

This study used a descriptive, exploratory qualitative design. The BR was selected as the study setting because of its significance within contemporary European migration patterns and the strong presence of civil society organisations providing health-related support to POTM. To capture different stages of the migration trajectory, the study included neighbouring countries along the route, countries of first arrival and transit (Türkiye and Greece), and destination countries (Austria and Germany). CSOs were identified through an online search using Google, the Worldwide NGO Directory [42], and the Platform for International Cooperation on Undocumented Migrants member list [43], based on the UN definition of CSOs [25] and activity in health support along the BR. This yielded 67 CSOs across 12 countries: Türkiye (*n* = 6), Greece (*n* = 7), Albania (*n* = 6), North Macedonia (*n* = 3), Serbia (*n* = 10), Kosovo (*n* = 2), Bosnia and Herzegovina (*n* = 6), Bulgaria (*n* = 5), Croatia (*n* = 5), Slovenia (*n* = 2), Austria (*n* = 4), and Germany (*n* = 11). Most were international organizations operating across multiple countries (*n* = 36), followed by national organizations (*n* = 18), national branches of international organizations (*n* = 8), and regional or local organizations (*n* = 5).

Study participants were individuals who had worked, in paid or voluntary roles, for CSOs providing health-related support to POTM along the BR within the past ten years. “Health support” included medical care, psychosocial assistance, referrals, healthcare-related legal advice, and other support enabling access to the right to health. Individuals involved only in auxiliary tasks (e.g., transport, data entry) or unable to communicate in English or German were excluded.

The recruitment process combined purposive and maximum variation sampling to ensure relevance and diversity across countries and organizational levels. Initially, 15 CSOs were selected and contacted via official email addresses. Responding organizations received the interview questions in advance, and interviews were conducted with relevant staff (e.g., coordinators, healthcare providers, project managers). After open coding of the first eight interviews, 14 additional CSOs were contacted based on their potential thematic contribution. Of the 29 organizations approached, 13 responded, with 10 nominating participants; three declined due to lack of relevant expertise. Additionally, five participants were recruited through snowball sampling across countries and CSOs, resulting in a total of 15 semi-structured interviews. Recruitment ended once at least one participant had been interviewed from each target country (except Albania, due to no response), with additional interviews conducted in selected countries to capture variation and achieve data saturation. Data saturation was jointly assessed by the first two authors.

### Data collection

Data were collected through semi-structured expert interviews with professionals working in CSOs providing support to POTM. Participants were interviewed in their professional roles to draw on their specialised knowledge and experience, consistent with the characteristics of expert interviews described by Bogner et al. [44].

The interview guide was developed based on the existing literature on migration health, POTM, and the role of CSOs, and refined with input from a co-author with extensive professional experience in providing health-related support to POTM through CSOs. Following two pilot interviews, the guide was revised; both pilot interviews were retained in the final dataset because of the richness of the information provided.

The final interview guide began with introductory questions and covered key topics including the role of CSOs in healthcare provision for POTM, challenges encountered in providing health-related support, opportunities for improving healthcare access, and the roles of different stakeholders. Follow-up questions and a checklist were used to ensure consistent coverage across interviews while allowing participants to elaborate on issues they considered important. An overview of the interview topics is presented in Table 1, and the complete interview guide is provided in Supplementary File 1.

Interviews were conducted between March and May 2024 via video call, audio-recorded, and lasted approximately 40 min. All interviews were conducted by the first author, with support from the second author during the first four interviews. Participants were invited to review their interview transcripts for accuracy and raised no objections.

### Data analysis

All interviews were transcribed verbatim in their original languages by the first author. Participants were assigned anonymized two-digit codes, and identifying information was removed.

Data were analysed using qualitative content analysis following Mayring and Fenzl [45] in MAXQDA 2022 [46]. Two members of the research team independently coded the first four transcripts and jointly developed an initial coding framework. Differences in coding were discussed until agreement was reached. One researcher subsequently coded the remaining transcripts, while the coding framework was iteratively refined through regular discussions with a second researcher. Categories and subcategories were developed inductively from the interview data and were regularly reviewed with the research team until consensus was reached. Existing theoretical and conceptual frameworks were not used to guide coding or category development. Instead, after the analytical framework had been developed inductively, relevant conceptual models were used during the interpretation of the findings to situate the results within the broader literature. Illustrative quotations were selected jointly; German quotations were translated into English, and identifiers were omitted where necessary.

To enhance trustworthiness, participants were invited to review and discuss preliminary findings. Three participated, and their feedback informed the interpretation.

**Table 1:** Main questions in the semi-structured interview guide

Main question
How is healthcare for POTM organized in the country where you work?
What gaps are there for POTM in the healthcare system of the country in which you work?
What challenges does your CSO face in supporting POTM?
How can the work of CSOs for health support for people on migration routes, such as the Balkan route, be improved and supported?
What should be done to better meet the health needs of POTM?

## Results

A total of 15 interviews were conducted; Table 2 presents key characteristics of the study population.

**Table 2:** Working background and experience of the participants

ID	Country	Type of organization	Professional background	Formal education related to migration	Volunteer/ paid	Tasks within organization	Years of experience	Interview Language
1	Austria	international	social sciences	yes	paid	organizational management, case management	6–10	German
2	North Macedonia	NI*	law	yes	paid	organizational management, advocacy, outreach	6–10	English
3	Austria	local	administration	no	paid	organizational management, advocacy	11–15	German
4	North Macedonia	NI	administration	no	paid	organizational management, advocacy, outreach	> 30	English
5	Slovenia	NI	migration management	no	paid	organizational management, case management, advocacy	16–20	English
6	Germany	local	medicine	yes	volunteer	organizational management, case management, outreach, medical care	1–5	German
7	Croatia	international	social work	no	paid	organizational management, case management, advocacy	16–20	English
8	Greece	national	medicine	no	paid	organizational management, advocacy	25–30	English
9	Kosovo	NI*	law	yes	paid	organizational management, case management, advocacy	11–15	English
10	Türkiye	international	psychology	no	paid	organizational management, psychosocial support	6–10	English
11	Türkiye	national	public health	no	volunteer	case management, outreach, medical care	6–10	English
12	Bulgaria, Serbia	international	medicine, law	no	paid	organizational management, advocacy, outreach	6–10	German
13	Türkiye	national	medicine	no	volunteer	advocacy, outreach	6–10	English
14	Germany	international	medicine	no	paid	organizational management, case management, advocacy, outreach, medical care	11–15	German
15	Bosnia and Herze- govina	international	social sciences	yes	paid	organizational management, advocacy, outreach	11–15	German

*NI = no information provided due to privacy concerns

**Fig. 1:**
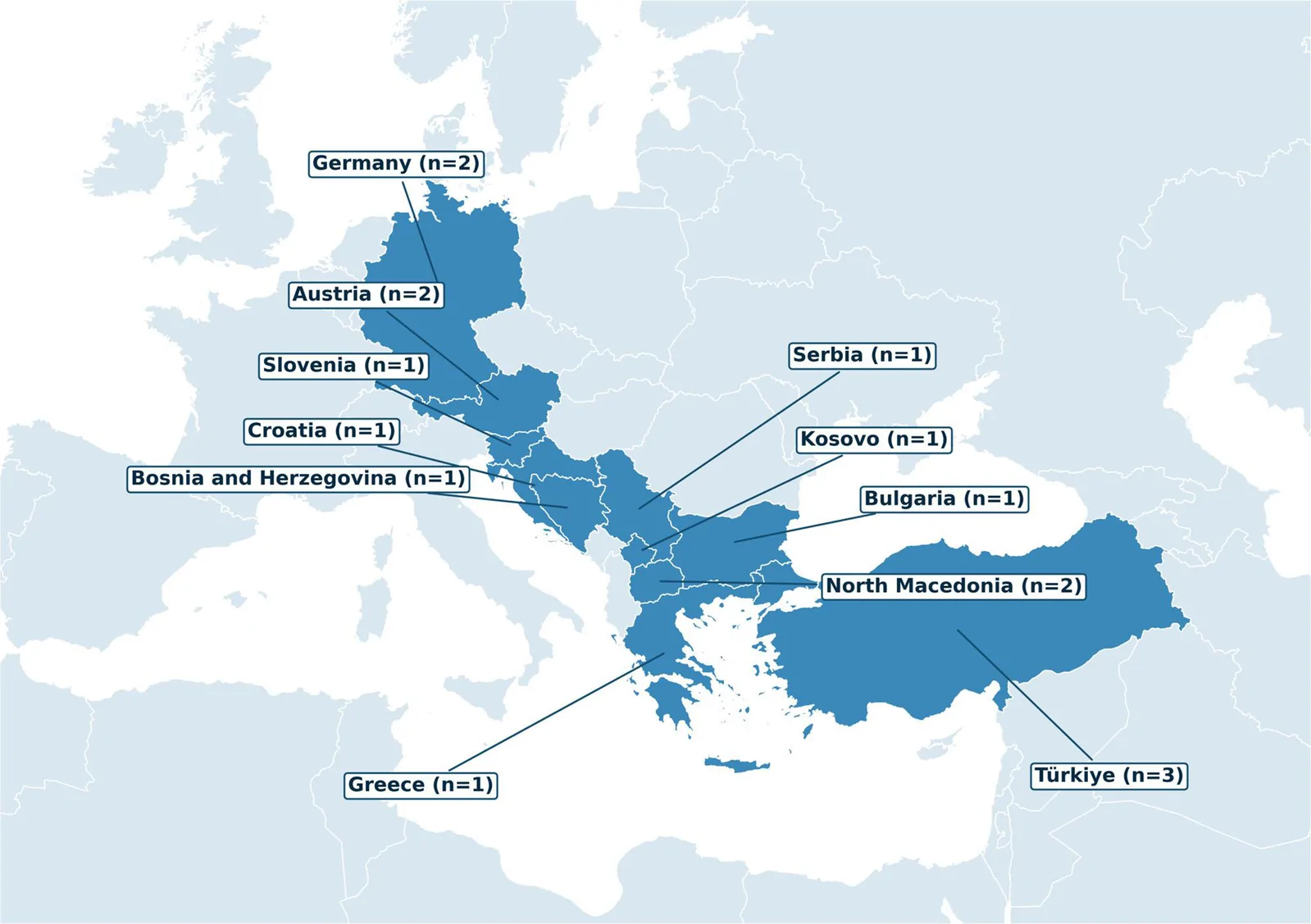
Countries represented in the study and number of interview participants. Note One participant worked in two countries and is therefore counted twice in the figure; the total number of participants was 15

The sample included CSO workers from local, national, and international organizations across 11 countries (Table 2; Fig. 1). Two international organizations were represented by participants working in different countries. Professional qualifications, as reported during the interviews, covered a diverse range of fields, including medicine, law, psychology, public health, social sciences, administration, and migration management. Five participants had formal education in migration-related fields completed at bachelor’s or master’s level, such as migration health or migration law, while the remaining participants had developed migration-related expertise through professional experience. Participants held diverse roles within their organizations, including organizational management, medical care, psychosocial support, case management, advocacy, and outreach to POTM. The sample comprised 12 paid staff members and three volunteers. Participants had between one and more than 30 years of experience working with POTM (mean 13.5 years; median 12 years).

**Fig. 2:**
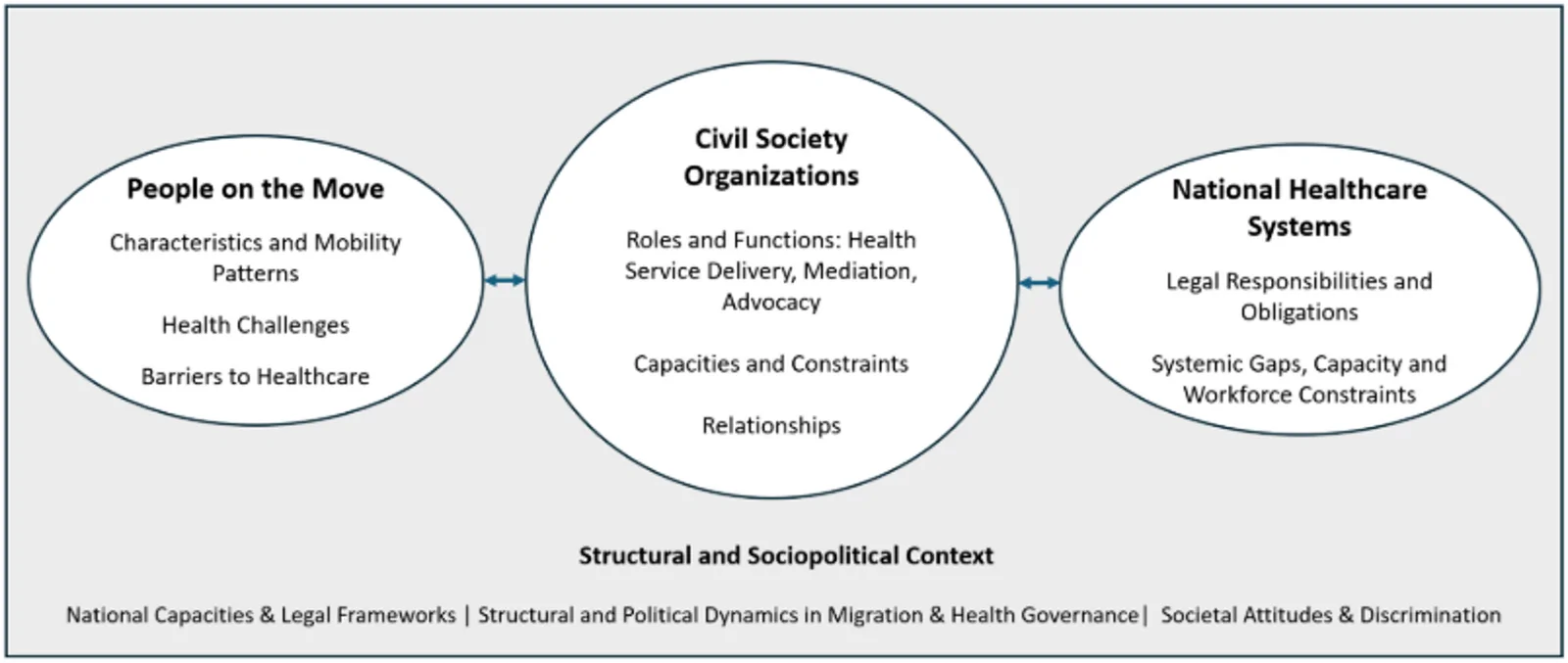
Analytical framework illustrating the relationships among the main categories identified through the qualitative analysis

The analysis generated four interrelated analytical categories (Fig. 2). The first category describes the structural and sociopolitical conditions shaping healthcare for POTM, including national capacities, legal frameworks, migration governance, and societal attitudes. The second focuses on POTM as the population served, highlighting their characteristics, health challenges, and barriers to healthcare. The third examines the role of national healthcare systems by contrasting their legal responsibilities with the structural and operational limitations that leave gaps in healthcare provision. Building on these contextual categories, the fourth examines how CSOs address the resulting gaps in healthcare provision through service delivery, mediation, and advocacy, while also exploring the capacities, relationships, and structural constraints that shape their ability to fulfil these roles.

### Category A. Structural and sociopolitical context

Structural and sociopolitical factors shaping CSOs’ health support included national capacities and legal frameworks, political dynamics and governance, and societal perceptions.

#### National capacities and legal frameworks

Participants described marked differences in national capacities to respond to the health and humanitarian needs of POTM. These capacities encompassed health system capacity, institutional preparedness, human and financial resources, and intersectoral coordination. Interviewees from former Yugoslav countries highlighted under-resourced public institutions, shortages of personnel and funding, and limited intersectoral coordination, which constrained the ability of health and social systems to respond effectively.

*Due to the capacities of the country to even be functional in this kind of crisis was almost impossible because as a country, we are also not very good in socio-economic sense. Therefore, usually the humanitarian asset of the country is fully provided by international actors. (ID-2, North Macedonia)*

One participant characterised this as a broader “system failure” affecting not only the healthcare system but also legal, social welfare, and economic structures, with international organisations filling humanitarian gaps. Participants from other countries also reported that increasing numbers of POTM strained resources and that better use of existing ones could ease pressure.

Although the right to healthcare was often formally guaranteed, participants described a gap between law and practice, with access largely dependent on migration status:

*It depends on the status. If they are asylum seekers, they have access through the asylum center. If they are in the detention center, the primary healthcare is through us and the healthcare is through the state system. POTM under subsidiary protection have the same access as the citizens. (ID-4, North Macedonia)*

While individuals granted protection were often integrated into national healthcare systems, those in transit, living in camps, or without regular migration status frequently had access only to basic or emergency care. More comprehensive healthcare access for eligible groups was reported in Austria, Germany, Kosovo, North Macedonia, and Türkiye, although practical barriers often limited access even where formal entitlements existed. By contrast, participants described access to healthcare for undocumented or unregistered people as being largely restricted to emergency care or NGO-supported services across several countries, including Bosnia and Herzegovina, Bulgaria, Germany, Serbia, Slovenia, and Türkiye. Participants further emphasized that access to healthcare depended primarily on legal status rather than country alone.

#### Structural and political dynamics in migration and health governance

Participants described migration and health governance structures as fragmented, bureaucratic, and poorly adapted to the needs of POTM.

*The problem is*,* from one side you do not provide services*,* on the other side you make them sick and third*,* you do not integrate them and there is a burden*,* there is a mess. (ID-8*,* Greece)*

Bureaucratic regulations, including data protection requirements, were described as major obstacles for CSOs, contributing to fragmented documentation systems. One participant suggested developing response blueprints for sudden influxes, noting their absence during arrivals from Ukraine in Germany.

Participants also described inter-ministerial coordination problems, such as licensing healthcare services in facilities governed by another ministry:

*What we are doing from some years now is trying to convince the Ministry of Health to license this space*,* so we can give the primary healthcare inside the center. But it is becoming a big deal*,* because according to the Ministry of Health*,* they cannot license a space inside another space*,* which is under the mandate of another ministry. (anonymous)*

Political factors were also described as undermining effective governance, with several interviewees perceiving EU and national governments as prioritizing border control and deportation over the rights of POTM:

*There is actually no fundamental will to give these people any life prospects or accommodation prospects*,* let alone medical care prospects. (ID-15*,* Bosnia and Herzegovina)*

Some participants also described migration as being used to divert attention from domestic issues, alongside “facade projects” that lacked resources or shifted responsibility onto CSOs and private actors. At the same time, a few interviewees noted that legal obligations were sometimes respected by institutions and individuals.

#### Societal attitudes towards POTM and discrimination

Discrimination emerged as a major barrier at individual, institutional, and societal levels, with participants describing widespread stigmatization of POTM in media and public discourse, often linking them to disease, violence, or so-called “health tourism.” Exclusionary practices within healthcare systems were evident:

*They didn’t allow the distribution of Kurdish language brochures. The state’s official ideology against Kurdish includes a restrictive policy. Yes*,* we saw that there*,* too. We didn’t have a problem with Arabic language*,* but we had trouble with Kurdish language brochures. (ID-13*,* Türkiye)*

Participants also reported that ethnicity could affect access to care and treatment quality, including cases in which doctors refused treatment or behaved inappropriately. However, participants also highlighted instances of solidarity, noting that local communities in Türkiye supported refugees despite hostile political rhetoric, while language barriers sometimes contributed to misunderstandings or perceived discrimination.

Some participants emphasized that public support could enable CSO work, influencing donations and policy, and suggested anti-racism education in schools and universities to reduce prejudice and improve awareness.

### Category B: People on the move

#### Diverse characteristics and mobility patterns

Interviewees consistently described POTM as a highly diverse group:

*They are not just men*,* it is a population*,* that is including women*,* children*,* elderly people*,* pregnant women*,* people with disabilities*,* which means*,* we have a variety of people and nationalities coming to the country. (ID-8*,* Greece)*

Participants also reported that border procedures carried out by police and European Border and Coast Guard Agency (Frontex) often isolated POTM and restricted contact with CSOs until individuals were either released or forcibly returned. POTM’s often short stays in transit countries further limited engagement with CSOs and healthcare systems, making sustainable treatment difficult.

Fear of being documented, detained, or deported was described as another major reason why POTM avoided seeking healthcare, even when medical needs were urgent:

*“If you need medical assistance*,* in most cases you are forced to come forward. This means you must give your fingerprints or identify yourself*,* so you can’t proceed unnoticed. This hinders your journey. That’s why so many people with medical needs are simply afraid to see a doctor.” (ID-12*,* Bulgaria*,* Serbia)*.

#### Health challenges before, during, and after migration

Interviewees described health challenges affecting POTM across the migration process. Many predated migration linked to trauma, weak healthcare systems, and limited preventive and dental care in countries of origin. The journey itself further worsened these conditions through exposure to violence and life-threatening events. Participants reported dehydration, exhaustion, and physical and mental health problems, sometimes leading to exclusion from shelter or basic services due to behavioural conflicts.

*Most of the time*,* what I have been seeing*,* medical issues are related and created by the living conditions. (ID-8*,* Greece)*

In transit and host countries, poor living conditions further increased health risks. Participants described overcrowded camps, inadequate hygiene, and limited access to care, heightening exposure to infectious diseases and violence. In some cases, lack of accommodation led to homelessness or onward movement. While most accounts highlighted inadequate conditions, one participant noted a well-equipped camp supported by EU funding.

#### Barriers to healthcare access

Interviewees identified legal status, financial constraints, and administrative barriers as key obstacles to healthcare access. Even where formal entitlements existed, access often depended on healthcare providers’ awareness and knowledge of migrants’ healthcare rights. Limited awareness could lead to treatment refusal, administrative complications, detention, or unexpected costs, while many POTM could not afford healthcare or medication.

Beyond these structural barriers, interviewees also described communication and information-related challenges as major obstacles to healthcare. Language and cultural differences often left both patients and providers unable to communicate effectively:

*That was the biggest problem*,* that communication is so crucial in medicine and often just wasn’t there*,* leaving one completely helpless because then there’s simply nothing you can do. (ID-6*,* Germany)*

Doctors were sometimes reported to refuse care due to linguistic or cultural barriers. Even when interpreters were available, translating medical documents and symptoms often complicated diagnosis and treatment. However, in North Macedonia, POTM from the Near and Middle East and African regions were sometimes reported to face fewer barriers due to greater cultural familiarity and similarities in healthcare practices.

Participants also discussed difficulties among POTM to navigate healthcare systems, including identifying providers, understanding bureaucratic procedures and knowing their rights. Limited health literacy regarding nutrition, medications, and basic bodily functions sometimes contributed to mistrust or refusal of interventions such as vaccinations. Education and guidance were therefore seen as crucial for improving both health outcomes and longer-term autonomy within healthcare systems.

### Category C: National healthcare systems

National healthcare systems were described in terms of how they provide care for POTM.

#### Legal Responsibilities and obligations

Participants emphasized that healthcare for POTM is a legal and institutional responsibility of the state:

*The government do not have a role*,* they have a responsibility. (ID 8*,* Greece)*

Responsibility was primarily attributed to national health authorities and public health systems, formally mandated to provide care for migrants and asylum seekers. However, participants described a gap between these obligations and their implementation in practice.

Reported access to healthcare varied considerably according to legal status, registration, and place of residence. Participants reported broader formal access to primary and specialist healthcare for registered asylum seekers and people granted protection in Austria, Croatia, Germany, Kosovo, North Macedonia, Slovenia, and Türkiye. In contrast, asylum seekers in Slovenia and Croatia, undocumented people in several countries, and people in transit, camps, or following pushbacks were often reported to rely on emergency, basic, or CSO-mediated care. Even where broader entitlements existed, access to secondary and specialized services frequently remained inconsistent because of administrative, financial, and capacity-related barriers. In some cases, these services became accessible only through informal professional networks or sustained CSO advocacy.

Participants also highlighted more inclusive models, such as a well-established Austrian program for uninsured individuals:

*In Austria*,* we offer the largest range of medical services for uninsured people*,* including specialists*,* general practitioners*,* a children’s consultation*,* vaccination advice*,* and vaccinations. (ID-3*,* Austria)*

Participants viewed such examples as evidence that more inclusive healthcare provision is possible when supported by political will and institutional commitment.

#### Systemic gaps, capacity and workforce constraints

Beyond implementation gaps, participants described structural constraints, including shortages in infrastructure, funding, personnel, and coordination. Interviewees from former Yugoslav countries reported particularly severe shortages, including limited availability of medications in hospitals:

*You would be lucky enough if you get the actual medicine. And [the original one] was sold on the black market. (anonymous)*

Where public systems failed, private hospitals were reported to provide better care but remained inaccessible to most POTM due to high costs, reinforcing socioeconomic inequalities.

Healthcare workforce shortages were often linked to the emigration of trained staff, described as “brain drain” in Southeast Europe. However, understaffing was not limited to resource-constrained settings; in countries such as Germany, especially in rural areas, long waiting times meant that even those with legal entitlements struggled to access timely care. Participants also highlighted administrative fragmentation, noting that weak coordination and limited foresight further exacerbated funding and staffing constraints.

### Category D: civil society organizations

CSOs play diverse roles in addressing the health needs of POTM, while structural and operational challenges affect their capacity to provide sustainable support.

#### Roles and functions of CSOs in migration health

##### Delivering and mediating healthcare

Interviewees described CSOs as a primary access point to healthcare services for POTM, especially those without legal status or facing administrative procedures. CSO services were described as essential, with some operating around the clock. Health-related support included arranging appointments, facilitating treatment, covering costs, translation, cultural mediation and informing both POTM and healthcare providers about rights and procedures.

*We can say that it is an in-between within the healthcare service and the migrant. (ID-11*,* Türkiye)*

Several interviewees described their organizations as engaging with healthcare providers, insurers, and government ministries, particularly when POTM needed services beyond formal entitlements. Beyond individual case support, CSOs conducted awareness campaigns, distributed information, and held workshops to help POTM navigate asylum and healthcare systems. Some also fundraised to cover medical costs, including surgeries. Participants also emphasized holistic care, including mental wellbeing and empowerment:

*It’s also very much about empowering the people who come to us… giving people a bit of dignity*,* humanity*,* acceptance*,* and recognition*,* and not seeing them merely as objects. (ID-14*,* Germany)*

##### Health advocacy

Beyond service provision, many CSOs engaged in advocacy through data collection, public statements, media engagement, and communication with policymakers.

*Because we are also a part of the state. We are taxpayers and at the same time we need to make the government accountable. (ID-8*,* Greece)*

Interviewees reported advocacy successes, including improved vaccine access, ending detention of mothers, and expanded public health coverage. Advocacy was seen as most effective when evidence-based.

##### The role dilemma: emergency support vs. state substitution

Interviewees described tensions around the expanding role of CSOs. While some saw them as temporary responders and advocates, others noted they had become de facto parts of healthcare systems by filling state gaps. In the absence of alternatives, many CSOs and staff felt morally compelled to act beyond their intended roles:

*We also had a fundamental principle: not remaining silent about any event we witnessed. (ID-13*,* Türkiye)*

Several participants questioned the sustainability of this expanding role and emphasized that CSOs should complement rather than replace public institutions:

*The ideal situation would be if institutions like our organization were not needed. (ID-3*,* Austria)*

Many viewed long-term solutions in integrating POTM healthcare into national systems, with CSOs focusing on advocacy, orientation, and rights protection rather than compensating for system failure.

#### Capacities and constraints of CSOs

While CSOs often filled critical gaps, interviewees were clear about the limits of their capacities and the challenges in providing sustained support.

##### Human resources

CSO teams ranged from large organisations with thousands of staff to small local volunteer groups. Most relied heavily on volunteer labour, often driven by strong moral commitment:

*Our staff is not only people who are paid to do something*,* but we are doing this because we have the heart to do this. (ID-9*,* Kosovo)*

Participants reported that emotional strain, high workloads, unstable employment, and low pay contributed to burnout and hindered recruitment and retention of specialised staff. Despite these constraints, some highlighted the value of culturally diverse, highly skilled teams with strong local knowledge.

*Local helpers […] just know better how to act in this third country*,* in this situation on the ground… This know-how is of incredible value to us. (ID-15*,* Bosnia and Herzegovina)*

Some CSOs offered training in law and migration, but mediation training, particularly for interactions with authorities or local communities, was often lacking. One interviewee also criticised the deployment of inadequately trained staff to high-pressure settings.

##### Financial instability

Interviewees consistently identified financial resources as a key limitation. Some CSOs declined government funding to maintain independence, while others depended on it for stability, yet funding was often short-term, project-specific, and unpredictable.

*It is difficult to use the money of donors in their budget format… They are giving lots of money for the meetings*,* but it is not easy to allocate that money to giving people in need what they need. (ID-11*,* Türkiye)*

- Some participants criticised donor priorities for visible emergencies over long-term needs, creating funding models that conflicted with CSOs’ ethical principles and hindered long-term planning, particularly for those excluded from public funding.

#### CSO relationships in migration health

##### Relationships with people on the move

Interviewees described trust as central to relationships with POTM and stressed the importance of safe environments for open discussion of needs:

*I would say our relation*,* our work*,* always starts based on trust. We know that we must provide a safe*,* comfortable environment for POTM to speak out. (ID-5*,* Slovenia)*

Participants noted that shared backgrounds and strong organisational reputations could build trust. Regular contact and consistency were also seen as key to strengthening relationships and facilitating access to services and administrative processes.

##### Relationships with local communities, healthcare systems, and public authorities

Interviewees described mixed relationships with local communities. While medical organizations were often viewed more positively, some reported limited awareness and hostility, including vandalism of CSO vehicles. Others described actively building trust with local communities:

*We have programs for the domestic population. We have a big trust*,* and they inform us if they found some vulnerable groups around the border regions. (ID-4*,* North Macedonia)*

Community support and visibility were also described as protection against political harassment.

Relationships with healthcare providers and institutions also varied considerably. Participants described collaboration with doctors, health ministries, and insurance providers, including data exchange and logistical support. However, dissatisfaction was frequently expressed regarding local health departments and limited institutional support.

*I was always in contact with the local health department, and I found it incredible how slow it was. It took so long, and they ultimately relied on us to just handle healthcare for POTM on the side. (ID-6, Germany)*

Participants also reported limited institutional support during the COVID-19 pandemic and little interest in training on the health rights of POTM. At the same time, some described improvements in cooperation over time, including access to governmental health databases.

Relationships with public authorities ranged from formal collaboration to active obstruction. While some collaborations were formalized through working groups, many participants described bureaucratic barriers and limited trust. Others reported political pressure, restrictions imposed by law enforcement, and limited access to POTM by actors such as Frontex.

*When I am trying to criticize my government*,* I am being blocked. For example*,* I will not be invited to certain meetings. (ID-8*,* Greece)*

Some participants described support from public authorities as being influenced by political considerations. Cooperation was more effective when CSOs had strong reputations, a long-term presence, formal partnerships, and close collaboration with local authorities.

## Discussion

CSOs are widely recognized as key actors in humanitarian response [24, 47, 48], and our findings illustrate why this role is indispensable along the BR. Healthcare access for POTM is shaped by legal, administrative, political, and societal barriers that extend beyond the capacities of healthcare systems alone. In response to these barriers, CSOs assume a dual role as both providers of health-related services and mediators between POTM, healthcare systems, and public authorities, thereby contributing to continuity of care and facilitating access to healthcare. Together, these findings underscore the importance of trust, collaboration, and clearly defined institutional responsibilities for equitable healthcare access.

Although geographically limited to the BR and based solely on the perspectives of CSO workers, this study provides broader insights into how healthcare access for POTM is shaped by the interplay between structural barriers, healthcare systems, public authorities, and civil society. These findings contribute to ongoing debates on the respective responsibilities of governments and CSOs in ensuring equitable healthcare access and highlight the structural conditions under which collaboration may facilitate or hinder access to care.

POTM face severe and often life-threatening health challenges as documented in prior literature [5, 9, 11–13] and consistently emphasized by our participants. Health vulnerabilities emerge before, during, and after migration and are further compounded by systemic, structural, and communicative barriers to healthcare. Their diversity, mobility, fear of detection, and limited contact with support systems further complicate access to healthcare and the delivery of healthcare services. At the same time, many countries along the BR face major healthcare resource constraints and often struggle to meet the needs of their own populations. In this context, international aid remains indispensable, as illustrated by the EU’s allocation of €115 million for humanitarian assistance in Southeast Europe and neighbouring regions in 2024 [49]. According to interviewees, CSOs are central to these efforts and remain essential in delivering healthcare to POTM, reflecting their broader contributions identified elsewhere [25, 47]. However, this also creates ambiguity regarding responsibility for meeting POTM’s health needs and raises broader questions about the respective roles of governments and CSOs in ensuring equitable access to healthcare.

### The evolving role of CSOs in migration health

Interviewees unanimously identified national governments and public healthcare systems as primarily responsible for providing healthcare to POTM. This aligns with international legal frameworks obliging states to address these needs [15, 19, 21, 22]. However, participants consistently emphasized that these obligations are often unmet in practice, reflecting a lack of political will. Similar patterns have been documented in Western Balkan countries before [50]. Accordingly, all interviewees reported that their organizations frequently compensate for these governmental shortcomings. This dynamic is not unique to the BR; similar findings were reported among grassroots CSOs in Germany during the so-called „Refugee crisis“ [51].

The interviews revealed three main ways CSOs support healthcare for POTM. First, they provide direct medical treatment, a role already documented in the literature [51, 52]. Second, they engage in advocacy, which has also been widely recognized [23, 24, 52]. Third, and more prominently emphasized by participants, they facilitate access to healthcare by arranging appointments, providing interpreters, offering financial support, and explaining procedures to both POTM and providers. While previous studies often subsumed these activities under broader service provision categories [24, 52], interviewees consistently described CSOs as bridges between POTM and healthcare systems. This interpretation is supported by Zihnioğlu et al. [53] and by Brenner et al. [51]. This mediating role is particularly critical in contexts of unequal access, where legal, administrative, and communication barriers systematically disadvantage POTM.

The fact that CSOs provide healthcare while also functioning as mediators highlights an ethical dilemma. As argued by Piccoli et al., when CSOs assume roles traditionally held by the state, they risk replacing rather than supplementing public healthcare systems [23]. This threatens long-term sustainability and may enable government withdrawal from legal and moral responsibilities. One interviewee described how their organization strategically withdrew services to pressure the state into action. A similar case occurred in Israel in 2007, when the Israeli Open Clinic closed after being overwhelmed, forcing public authorities to confront their inaction [54]. These examples illustrate the complexity of responsibility-sharing between states and civil society and highlight the challenges of ensuring equitable and sustainable access to healthcare POTM.

Our findings can be interpreted through conceptual models describing both the roles of CSOs and their relationships with governments. Consistent with Young’s framework [55], two of the roles identified in our study correspond to supplementary and adversarial roles. CSOs filled gaps in healthcare provision by providing direct health services while simultaneously advocating for improved healthcare policies and government accountability. Najam’s framework [56] further helps explain the diverse relationships between CSOs and governments observed in our study. Najam argues that government-CSO relationships vary according to the extent to which the two actors share common goals and preferred strategies. Consistent with this perspective, participants described relationships ranging from tensions related to government policies and institutional practices to constructive collaboration with individual actors within public authorities and healthcare systems who shared the goal of improving healthcare access for POTM. These findings support Najam’s argument that governments should not be regarded as homogeneous actors and that government-CSO relationships are best understood at the level of specific institutions and actors rather than governments as a whole [56]. These different forms of government-CSO relationships have important implications for healthcare access, as they shape coordination between public authorities and CSOs, the distribution of responsibilities, and ultimately the ability of POTM to access healthcare.

Beyond these insights, our findings highlight an additional role that is not fully captured by existing conceptual models. Participants consistently described CSOs as mediators facilitating access to healthcare by connecting POTM with healthcare providers and public institutions. Although the frameworks proposed by Young and Najam acknowledge that government-CSO relationships are dynamic and context-dependent, neither explicitly conceptualizes a relationship in which governments retain primary responsibility for healthcare provision while CSOs primarily facilitate access to these services. Furthermore, these models do not distinguish between interactions occurring at different institutional and hierarchical levels within government. Our findings therefore suggest that conceptual models of government-CSO relationships in migration health should also account for CSOs’ mediation role and for interactions occurring at different institutional and hierarchical levels. Recognizing this mediation role highlights that government-CSO relationships are not only a matter of governance but also a key determinant of equitable healthcare access for POTM.

Interviewees in this study, as well as Padilla et al. and Kentikelenis et al., emphasized that CSOs will likely become even more important in the future while lacking the capacity to fill gaps beyond acute emergencies [24, 52]. In this context, the mediator role appears particularly valuable. It allows CSOs to support healthcare access without relieving governments of their responsibilities, especially when paired with advocacy. It also requires fewer specialized resources than direct service provision and may therefore be more sustainable long term. Direct healthcare provision by CSOs may therefore be most appropriate in acute crises, while long-term efforts could focus on mediation and advocacy. Meanwhile, the international community could assess whether states require support in fulfilling their responsibilities toward POTM.

### Trust and collaboration for effective healthcare support

The findings of this study suggest that effective health support for POTM depends on trust and collaboration between CSOs, public authorities, and healthcare providers. Taken together, existing legal and institutional frameworks appear conducive to building such relationships. While international agreements such as the Universal Declaration of Human Rights [20] and the Refugee Convention [15] provide a general normative foundation for CSO activities, they do not regulate them directly. Within the EU, regulations and institutions enable CSOs to participate in political processes and receive support for social projects [57–59]. Similarly, the Council of Europe promotes legal frameworks that safeguard CSO independence and participation in public decision-making [60]. Despite these supportive legal and institutional frameworks, our findings and previous studies indicate that CSOs continue to encounter major barriers, including criminalization, restrictive legal and administrative measures, reduced funding, and limited governmental cooperation [61–64]. Recent evidence from across the EU further documents the increasing criminalisation of solidarity, including judicial proceedings, administrative sanctions, and other forms of harassment targeting individuals and organizations supporting migrants [43]. These findings are consistent with the concept of hostile hospitality proposed by Schack et al. [65], who argue that governments may perceive CSO activities as challenging migration governance strategies and respond by seeking to retain control over the provision of support. Such dynamics may undermine the trust and collaboration between CSOs, public authorities, and healthcare providers that are essential for ensuring effective health support for POTM.

Our findings further suggest that trust and collaboration between CSOs and public authorities can be strengthened through sustained interpersonal relationships, formalized collaboration mechanisms, and respect for CSO independence. Effective collaboration was most reported at lower administrative levels, where direct personal contact enabled trust to develop over time. Formalized collaboration mechanisms also appeared beneficial, although cooperation often remained dependent on personal initiative and was insufficiently coordinated, consistent with the findings of Brenner et al. [51]. At the same time, participants emphasized that maintaining CSO independence was essential to avoid becoming overly aligned with governmental agendas, a concern also highlighted in previous studies [66, 67]. This balance between collaboration and independence should therefore be considered when implementing proposals, such as those by Kövér and the Prague Security Studies Institute, advocating stronger government–CSO partnerships [68, 69]. Developing collaborative frameworks that foster trust and coordination while safeguarding CSO independence may therefore strengthen collaboration without compromising the advocacy role of CSOs. Ultimately, such collaboration may improve coordination between public authorities, healthcare providers, and CSOs, thereby facilitating equitable healthcare access for POTM.

Trust also shapes relationships between CSOs and the broader population, with important implications for healthcare access for POTM. Previous studies have shown that public trust enables CSOs to mobilize donations, volunteers, and broader societal support for their activities [70–72]. Conversely, prejudice and mistrust may reduce public support and constrain CSOs’ ability to deliver health-related services and facilitate access to healthcare [53]. While some authors attribute such scepticism to limited accountability of CSOs, the determinants of public trust appear to be more complex [73, 74]. Consistent with these findings, participants in our study who actively engaged with local communities generally reported stronger relationships and greater public acceptance. Strengthening relationships with local communities may therefore enhance both public support for CSOs and their ability to facilitate equitable healthcare access for POTM.

### Strengths and limitations

A major strength of this study is its cross-country qualitative design, which enabled the exploration of CSOs’ experiences across diverse migration and healthcare contexts along the BR. Including participants from different types of CSOs and professional backgrounds provided a broad perspective on healthcare provision for POTM and the challenges faced by CSOs. By examining multiple countries along a shared migration corridor, the study provides insights into common regional patterns across diverse national contexts. However, the findings should be interpreted within the scope of the BR. Although this corridor represents an important European migration pathway, its structural, legal, and policy context may differ substantially from those of other migration routes, particularly maritime routes. Therefore, the transferability of the findings to other migration contexts should be considered with caution [75].

Several methodological limitations should also be considered. Despite efforts to ensure a diverse sample, only CSOs that responded positively to interview invitations could be included. Therefore, we cannot claim to represent the full range of CSOs operating along the BR. Volunteers and frontline workers directly involved in healthcare provision may also be underrepresented because of language barriers or limited availability, and their perspectives could have provided additional insights. Furthermore, reporting bias cannot be excluded, as participants may have downplayed organizational challenges or presented their work more favourably. In addition, although participants frequently referred to national laws, healthcare entitlements, and administrative regulations, these accounts were not systematically cross-checked against legal or policy documents. The findings therefore reflect participants’ understanding of the legal and institutional context rather than a legal analysis of national frameworks. Future research could build on these findings through field-based, context-specific studies, as suggested by van der Borgh et al. [76], as well as mixed-methods approaches.

## Conclusions

States bear the primary legal responsibility for ensuring equitable access to healthcare for POTM. Nevertheless, this study demonstrates that CSOs play an indispensable role in addressing the healthcare needs of POTM along the BR. Our findings show that CSOs not only provide direct health services and advocate for more equitable policies but also act as key mediators by connecting POTM with healthcare providers and public authorities, thereby facilitating access to healthcare. Our findings highlight the mediating role of CSOs in facilitating healthcare access by helping overcome legal, administrative, and communication barriers while supporting states in fulfilling, rather than replacing, their responsibilities. At the same time, CSOs face persistent operational barriers, including funding shortages, workforce constraints, trust-building challenges, language barriers, and adverse political and societal environments. Despite international recognition, they continue to face criminalization, restrictive regulations, and exclusion from decision-making processes, all of which constrain their ability to support equitable healthcare access.

As a qualitative case study based on CSO perspectives along a single migration route, this research provides important insights but does not capture all dimensions of healthcare provision for POTM. Future research should examine how trust and collaboration can be strengthened among CSOs, governments, healthcare providers, host communities, and POTM to improve coordination and equitable healthcare access while preserving CSO independence. It should also explore how the mediating role of CSOs can be strengthened within different models of government-CSO collaboration while maintaining their capacity to act as independent advocates within equitable and accountable health systems.

## Supplementary Information

Below is the link to the electronic supplementary material.

Supplementary Material 1

Supplementary Material 2

## Data Availability

No datasets were generated or analysed during the current study.

## Declarations

### Use of artificial intelligence

Generative AI was used to support English language editing and to assist in preparing the map presented in this manuscript. The authors take full responsibility for the content of the manuscript.

## Abbreviations

BR: Balkan Route
CSO: Civil Society Organization
EU: European Union
Frontex: European Border and Coast Guard Agency
POTM: People on the Move
UNHCR: United Nations High Commissioner for Refugees
