# Supplementary material for: Health support for people on the move: a qualitative exploration of civil society organizations’ roles and obstacles along the Balkan route
Source: Int J Equity Health. 2026 Sep 24;25:222. doi: 10.1186/s12939-026-03048-x (PMC13613670; doi:10.1186/s12939-026-03048-x)
Supplement: Supplementary file 1 [file 12939_2026_3048_MOESM1_ESM.docx]

**Interview Questions**

1. Please introduce yourself and your organization.

- Professional background and role
- Organization type and main activities
- Experience working with people on the move

2. What is your organization's role in providing healthcare for people on the move along the Balkan Route?
3. Based on your experience, what are the main health needs and health problems of people on the move?
4. How is healthcare for people on the move organized in the countries where you work?

- Main actors and responsibilities
- Differences across settings or legal status

5. What are the roles of governments and civil society organizations in providing healthcare?

- Gaps in the healthcare system
- Division of responsibilities

6. What are the main challenges your organization faces in supporting people on the move?

- Legal or administrative barriers
- Strategies for addressing these challenges

7. What factors facilitate your organization's work?
8. How could support for civil society organizations be improved?

- Collaboration between CSOs and governments
- Examples of good practice

9. What changes are needed to better meet the health needs of people on the move?
10. Are there other organizations or actors working along the Balkan Route that play an important role in healthcare?
