# Supplementary material for: Health support for people on the move: a qualitative exploration of civil society organizations’ roles and obstacles along the Balkan route
Source: Int J Equity Health. 2026 Sep 24;25:222. doi: 10.1186/s12939-026-03048-x (PMC13613670; doi:10.1186/s12939-026-03048-x)
Supplement: Supplementary file 2 [file 12939_2026_3048_MOESM2_ESM.docx]

**Supplementary 2: Category Guide**

| **Category**  **-**  **ID** | **Category level (Main, Sub1, Sub2, Sub3)** | **Category**  **Name** | **Definition** | **anchor examples** | **coding guideline** |
| --- | --- | --- | --- | --- | --- |
| 1 | Main | Structural and sociopolitical context | Segments that describe the contextual factors which civil society organizations are working within. (legislative, socio-political, capacities, Balkan route, people on the move) | / | This category only serves as umbrella term for sub-categories. No segment is actually assigned ID 1. For actual categories and potential distinctions from other categories refer to sub-categories of ID 1. |
| 1.1 | Sub 1 | host country | Segments that describe contextual factors related to the host country such as financial resources, legislation and processes relevant for migration and health access for people on the move and socio-political attitudes and tendencies regarding people on the move, health care and civil society organizations. | / | This category only serves as umbrella term for sub-categories. No segment is actually assigned ID 1.1 For actual categories and potential distinctions from other categories refer to sub-categories of ID 1.1 |
| 1.1.1 | Sub 2 | capacity host country | Segments that describe the capacities of the host country to deal with migration procedures, accommodation and other aspects of migration other than health. | "However, due to the capacities of the country to even be functional in this kind of crisis was almost impossible because as a country, we are also not very good in socio economic sense." | This category only describes capacities not related to the healthcare system. Capacities of the healthcare system itself are coded with 4.2 (capabilities (of the healthcare system)) |
| 1.1.2 | Sub 2 | concepts and legislation | Segments that describe laws, procedures, and concepts relevant to migration and health of people on the move within the host country. | "Yes, even in a sense of policy making, there are so many things, that need to change legislatively." | This category only describes very general segments. Detailed segments on concepts, procedures, laws or inequalities regarding health are coded with sub-categories of 1.1.2 |
| 1.1.2.1 | Sub 3 | health support concept | Segments that describe how the state as a system looks at health of people on the move and approaches this issue. | "The problem is, from one side you do not provide services, on the other side you make them sick and third, you do not integrate them and there is a burden, there is a mess." | Themes that can be coded with this category include for example, is it holistic, fragmented or non-existent. |
| 1.1.2.2 | Sub 3 | health and migration procedures and policies | Segments that describe concrete procedures and policies that are followed in the realization of asylum, migration and access to health, including regulations for civil society organizations. | "So, the law foresees to have the health service but it does not foresee the regulation of the center, does not foresee to have extra staff, that works as a doctor." | Laws concerning access to health for people on the move are coded as 1.1.2.3 (access laws and services). For further details on the distinction of the two categories refer to 1.1.2.3. |
| 1.1.2.3 | Sub 3 | access laws and services | Segments that describe laws granting or not granting access to health for people on the move (undocumented, documented, protection status or similar) and the associated services available for those people. | "In a way, on the other side, I can tell you, that the reality of addressing the needs of people on the move or migrant, refugees or asylum seekers or what you want to name, it is related also how, for example, legal frames placed in the country. What does it mean access into a system and because it also has been changing, from 2016, for example, when everybody was allowed to access healthcare, then in 2019 this was changed, temporary access or emergency response." | This category is to be coded if the segment describes legal frameworks concerning access or the services available in theory, if the segment describes procedures, step by step regulations or rules how to implement a law code 1.1.2.2 (health and migration procedures and policies) is to be used. |
| 1.1.2.4 | Sub 3 | inequalities | Segments that describe differences of access to healthcare and related procedures within people on the move or between people on the move and the general population. | "There was a letter on the refugees' website, written by an Iranian refugee. He said that back then, there were no rights like those Syrians benefit from. They couldn't even go to hospitals properly. He said, "We want to benefit from the rights Syrians benefit from." | This category can overlap with 1.1.2.3 (access laws and services). This cannot be avoided entirely but the difference between both categories is that 1.1.2.3 focuses on the law/service itself and 1.1.2.4 focuses on the fact that there is a difference between groups. |
| 1.1.3 | Sub 2 | socio-political atmosphere | Segments that describe the socio-political environment. Such as the atmosphere in the population, government attitudes and preferences and resources allocation. | "What the Mayor of Konak told us, and especially what a woman told us was this: "We don't want to make Izmir an attractive place for refugees. So, we don't accept your request." That's their perspective on this matter." |  |
| 1.1.3.1 | Sub 3 | political and resource focus | Segments that describe where the government and the system focus their attention and resources on, including believes and attitudes of the government. | "But someone who works in Ministry2, they said, yes, I agree with you, but I cannot make those decisions. The minister is, who has to make it. But on the higher level, there is no will to change some things." | Segments that do not explicitly address the focus of the government or resources but describe behavior of the government that is interpreted as insufficient are coded as this category as well. |
| 1.1.3.2. | Sub 3 | adherence to law and responsibilities | Segments describing whether actors adhere to their legal duties in migration and healthcare support including comments on corruption and strategies the government employs to avoid responsibility | "Because if anything happens, the government will not take responsibility. And when it does happen, it's usually, you know, you figure this out and leave us out of the discussion." | This category is distinct from 1.1.3.1 (political and resource focus) in the sense that segments coded here describe active neglect of duties. Since corruption is reducing the capabilities of the healthcare system to fulfill its duties, comments on corruption are coded with this category as well. |
| 1.1.3.3 | Sub 3 | public opinion | Segments that describe the opinion and interest of the public/population and the media on and in (health support for) people on the move including its importance and effects. | [...] They [a governmental office] are like looking for quite few years for new places, where there could be new asylum centers around Slovenia. But then there is a protest from people who live there, the inhabitants of the town or whatever. |  |
| 1.1.3.4 | Sub 3 | stigmatization and racism | Segments describing racism, stigmatization, discrimination and prejudices against and towards people on the move. | "The main problem would be like an atmosphere in Slovene society promote. Yeah, I would say promoting anti migrant feelings let's say, like the migrants are responsible for everything, violence for, I don't know, sexual abuse, whatever." |  |
| 1.2 | Sub 1 | Balkan Route | Segments describing the characterizes of the Balkan Route including whether a country is an arrival, transit or destination country, its effects on civil society organizations´ work. | "Maybe it's a bit more easy for us to work with all the categories, because we don't have huge numbers of people arriving in Kosovo. I think you already know that Kosovo is only a transit country. It's not a destination country." |  |
| 2 | Main | CSOs | Segments that describe civil society organizations and different aspects of them like their work, roles, capabilities, workers and reputation. | / | This category only serves as umbrella term for sub-categories. No segment is actually assigned ID 2 For actual categories and potential distinctions from other categories refer to sub-categories of ID 2 |
| 2.1 | Sub 1 | functions | Segments that describe different aspects of the work of civil society organizations like their roles and the services they offer. | "I think the, in the most of the situation, the civil society organization or activist group or whatever include, they been taking the other, the difficult part of the so-called people, who are not-existing or are invisible for us." |  |
| 2.1.1 | Sub 2 | role description | Segments describing how civil society organizations see their own work on a meta level including their view of their future role. | "I hope, that there will not be the necessity of NGOs to provide some basic services that should be in place from the government." | Concrete descriptions of the work of civil society organizations is to be coded with 2.1.3 (advocacy), 2.1.4 (mediation) or 2.1.5 (provision). |
| 2.1.2 | Sub 2 | approach to health | Segments describing how civil society organizations look at health and approach it accordingly. | "As an organization we approach health as it is being defined by WHO, as a state of physical health, mental health and the well-being. I think what we are trying to approach different components and that is sometimes, maybe it is missing directly the so-called having a stethoscope or giving a tablet to a patient. But if you are supporting other parts that are contributing to health, like mental health, the well-being of people, food et cetera." |  |
| 2.1.3 | Sub 2 | advocacy | Segments that describe that civil society organizations advocate for the health of people on the move (addressing society or institutions) including remarks about how they hold institutions accountable. | "We do focus on advocacy, and we try to lobby for changes of the legislation to be more open, especially for foreigners." | This category includes also actions that cannot be seen as advocacy but serve the purpose of advocacy or holding institutions accountable. |
| 2.1.4 | Sub 2 | mediation | Segments describing the ways civil society organizations support people on the move access the healthcare system, treatment and medication. | "We are filling a gap just in this accessing the health services, actually more as anything else." | Work with the healthcare system aiding the access of people on the move is to be coded with this category as well. Overlap with 7.2 ((relationship) with the healthcare system) is possible. Actual healthcare provision through civil society organizations is coded with 2.1.5 (provision). |
| 2.1.3.1 | Sub 3 | organizing and accompanying access | Segments that describe how civil society organizations support people on the move by organizing appointments, referring to health related offers, filing applications for assumption of costs or transporting and accompanying them to appointments. | "Two or three people take charge of organizing the appointment with the person, finding interpreter support, taking them to the hospital." |  |
| 2.1.3.2 | Sub 3 | informing and integration | Segments that support how civil society organizations help people on the move through integration and informing them or healthcare providers about health in general, procedures and their rights. | "We always have to explain to doctors and nurses, no, they don't have insurance, they have right as Croatian citizen, but only with ID and then ministry will pay every costs." | If the comment focuses on the informing work of civil society organizations this category is to be used, if the comment focuses on the (degree) of expertise/knowledge of healthcare 5.5 (provider) is to be used. |
| 2.1.3.3 | Sub 3 | information and culture exchange | Segments describing how civil society organizations support people on the move through facilitating mutual understanding and the exchange of information between them and healthcare providers. | "We can say that civil society organizations, Organization3 also, giving translator services, translation support to migrants. It is one of the most important support, I think." | This category includes the provision of translator services and cultural mediation. Overlapping with 5.1 (culture and language) is possible, but in general, comments focusing on the role as mediator should be coded with 2.1.3.3, comments focusing on language and cultural problems should be coded with 5.1 |
| 2.1.3.4 | Sub 3 | financing treatment | Segments describing how civil society organizations support people on the move through financing their treatments. | "We organized the provision of medications for these individuals by setting a budget for everyone as much as our resources allowed" |  |
| 2.1.5 | Sub 2 | provision | Segments describing the direct provision of healthcare for people on the move through civil society organizations. | "And we have our medical unit in the detention center, and we are responsible there for the primary healthcare. We have a medical doctor three times per week." | Provision of medicine/drugs, tooth brushes, soap or other means of taking care of hygiene or one´s own health are coded with this category as well. |
| 2.2 | Sub 1 | capabilities | Segments that qualitatively or quantitively describe the capabilities/resources of civil society organizations to meet the needs of people on the move. | "We do not aim to be everywhere in that sense. So more to do as much as we can. The best we can, but not to, in a way, living like an illusion that you can help everyone and it is something that is not effective in, humanitarian work." | This category also includes comments on the amount of work or the desired amount of work done and way to support civil society organizations. |
| 2.2.1 | Sub 2 | human resources | Segments describing the workers of civil society organization in various aspects. | "As an office we are a very small team, we are only four, we used to be two, now we are four. Of course, we have some service contracted persons to support the work, that we do, but we are four staff." |  |
| 2.2.1.1 | Sub 3 | motivation and incentives | Segments that describe the motivation of civil society organizations´ workers or civil society organizations as a whole to be involved in health support for people on the move. | "Our staff is not only people who are paid to do something but we are doing this because we have the heart to do this." |  |
| 2.2.1.2 | Sub 3 | working conditions | Segments that describe the working conditions, such as work load, salary and safety of civil society organizations´ workers. | "It's [the] workload, that every employee has. It's like too much for one person." | Comments referring to the quality of the task description of individual civil society organization workers are coded as this category as well. |
| 2.2.1.3 | Sub 3 | shortages and changes | Segments that describe the adequacy of worker number including obstacles for civil society organizations due to worker number or recruitment. | "For example, in the reception center for asylum seeker there is a lack of staff. We have only two persons, who are providing assistance, health assistance, and we have only two cars on 15 other employees. We have a lot of activities. So, sometimes we don't have enough staff to cover all assistance." | Comments on the number of workers without addressing whether this number is appropriate/sufficient or not are to be coded as 2.2.1 (human resources). Since worker shortages/changes in the asylum system complicate civil society organizations´ work as well those are coded with this category as well. |
| 2.2.1.4 | Sub 3 | competencies and training | Segments that describe the competency and experience of workers (to deal with the tasks they are facing) and training they lack or might receive in that matter. | "When you come to the organization you have actually trainings on the field of voluntary work and then also in the field of migration." | Segments that only describe professions are not coded with this category. |
| 2.2.1.5 | Sub 3 | volunteers | Segments that specifically address volunteering. | "All other people working there are on a voluntary basis. So, all the doctors, additional nurses and such are on a voluntary basis." |  |
| 2.2.2 | Sub 2 | financial resources | Segments that describe aspects of the finances of civil society organizations such as sources, need for it and raising money. | / | This category only serves as umbrella term for sub-categories. No segment is actually assigned ID 2.2.2 For actual categories and potential distinctions from other categories refer to sub-categories of ID 2.2.2 Segments linking money to the sustainability of the work of civil society organizations are coded with 2.2.3.1 |
| 2.2.2.1 | Sub 3 | raising money | Segments that describe the process of raising money, specifically, associated struggles. | "But it is difficult to use them in their budget format. It is for me another obstacle. Because they are giving lots of money for the meetings but it is not easy to allocate that money, to giving people in need, what they need for the, for example, drugs and the other stuff." |  |
| 2.2.2.2 | Sub 3 | need for money | Segments describing the need for money. | "Each year you have a gap or issue with covering your border staff, as since 2017 they are not a lot of much of funds, which will enable us to, to fully provide services for everyone on a daily basis. So, we have to pick and choose what we can do." |  |
| 2.2.2.3 | Sub 3 | sources | Segments describing through which sources civil society organizations finance their work. | "As a humanitarian organization we are also based on donations, not only research grants or grants in general." |  |
| 2.2.3 | Sub 2 | sustainability | Segments addressing the sustainability of civil society organizations´ health support for people on the move. |  | This category only serves as umbrella term for sub-categories. No segment is actually assigned ID 2.2.3 For actual categories and potential distinctions from other categories refer to sub-categories of ID 2.2.3 |
| 2.2.3.1 | Sub 3 | money | Segments linking money to the continuity of civil society organizations´ work. | "Since we are project-based organization then it becomes very challenging, when the projects are going to end and we are in this situation. Our project will be ending in October this year so we need to prepare other proposals and to see if Organization2 will be able to support us again." | For distinction from 2.2.2 (financial resources) see comments of 2.2.2 |
| 2.2.3.2 | Sub 3 | processes | Segments linking processes of migration, health bureaucracy in healthcare support and humanitarian aid to the sustainability of civil society organizations´ work. | "It's not like, once we had this dentist, he can do checkups well or whatever or the diabetes, which is quite often. It's not like, okay, we can go there and we'll get this for the person. It's like, really doing all every time from scratch for the individual case." | Overlapping with other categories such as 2.2.1.3 (shortages and changes (in human resources)) or 1.1.2.2. (health and migration procedures and policies) cannot be avoided. Sustainability is an important concept which justifies this in the opinion of the author. |
| 2.2.3 | Sub 2 | reputation | Segments describing the reputation of civil society organizations among the public system, people on the move or society (as resource). | "With the refugees and migrants, you know, we have a good communication because [they] recognize the [...] logo like a very strong logo, who will support them." | Overlapping with 7 (relationships) can occur in some cases. |
| 2.2.4 | Sub 2 | number of patients | Segments describing the number of patients and the ability of civil society organizations to manage those. | "Maybe it's a bit more easy for us to work with all the categories, because we don't have huge numbers of people arriving in Kosovo." |  |
| 2.2.5 | Sub 2 | internal organization | Segments that describe how civil society organizations organize themselves internally. | "And it was a big challenge for us in the beginning to establish a system. Now it's a little bit better. And normally we have a system and structure because we are a little bit more experienced." |  |
| 3 | Main | POTM | Segments that describe characteristics of people on the move in regards of demography, health status/needs and risk factors. | / | This category only serves as umbrella term for sub-categories. No segment is actually assigned ID 1.2.2 For actual categories and potential distinctions from other categories refer to sub-categories of ID 1.2.2 |
| 3.1 | Sub 1 | Characteristics and mobility patterns | Segments describing demographic characteristics of people on the move (that get in touch with the civil society organization) including nationality, age and gender. | "Imagine that you have the population that is not just men, it is population that is including women, children, senior citizen, pregnant women, handicapped people, means we have a variety of people, you have a variety of nationalities coming to the country." |  |
| 3.2 | Sub 1 | health status and needs | Segments describing the health status and health needs of people on the move including comments on gaps in meeting those needs. | "So ultimately, when you get a person migrating from war or from different difficult life conditions, you already have a person with big trauma. Which is not addressed." | This category includes comments on learning disabilities, injuries and overall physical status as well. |
| 3.3 | Sub 1 | risk factors | Segments describing factors endangering people on the move´s health. | "And overcrowding means the same time, you are lacking resources, meaning you do not have enough food, you do not have enough shelter, you do not have enough heating, you do not have enough water and you do not have enough an access to healthcare." | Since most comments concerning accommodation are linking accommodation to bad living conditions and directly or indirectly to worse health outcomes general comments on accommodation including bad infrastructure around camps are coded using this category as well. |
| 4 | Main | national healthcare systems | Segments that describe national healthcare systems and different aspects of them like their work, roles and capabilities. | / | This category only serves as umbrella term for sub-categories. No segment is actually assigned ID 4 For actual categories and potential distinctions from other categories refer to sub-categories of ID 4 |
| 4.1 | Sub 1 | assignment | Segments describing the work of the healthcare system including its roles, shortcomings, expectations towards them and capacities. | "So, it is, I think, we are doing a lot of things, the government is doing things, but they still need to improve on providing everything." | Overlapping with 1.1.2 (legislation, organization, concepts) and 1.2.2.2 (health status and needs) can occur. Health services provided by public institutions other than clinics care coded with this category as well. |
| 4.1.1 | Sub 2 | role attribution | Segments that include the legal and subjective assignment of roles and responsibilities to the healthcare system and the actions derived from those roles. | "Ok, governments have a role, they do not have a role, they have a responsibility. Everything we talk about at the moment is a state responsibility." |  |
| 4.1.2 | Sub 2 | special services | Segments that describe special health services (not) provided by the healthcare system. | "And then all those who need secondary level of healthcare, they are immediately transferred to the hospitals in City1. And again, the medical treatment and everything gets done by our medical system, domestic medical system." |  |
| 4.1.3 | Sub 2 | financing services | Segments describing the government (not) financing services. | "Together with the ministry we were able to receive a fund from the ministry of health, to receive a fund to pay the heart surgery of the baby in City2, in Macedonia, in this private hospital which is very famous and very good." | This category includes comments on the financing of services not provided by the healthcare system itself as well. If the comment is about the government paying for services not included in the current system this category is to be used. If the comment is about regular services 4.1.2 (special services) is to be used. |
| 4.2 | Sub 1 | Gaps, capacity and workforce constraints | Segments describing the capability of a national healthcare systems to meet the health needs of people on the move and the domestic population. | / | This category only serves as umbrella term for sub-categories. No segment is actually assigned ID 4.2 For actual categories and potential distinctions from other categories refer to sub-categories of ID 4.2 |
| 4.2.1 | Sub 2 | quality | Segments describing the general quality of healthcare systems including services (not) available for domestic people. | "When it comes to healthcare in Macedonia, generally speaking, the health system is quite out of date and it's really not good. By that I mean almost completely crushed. We do have a lot of issues in the healthcare system in general speaking." | Overlaps with 1.1.3 (socio-political atmosphere) and 1.1.2.3 (access laws and services) are possible. If resource problems for domestic people such as shortage of workers or a lack of appointments are described 4.2.2 (resources) is to be used. |
| 4.2.2 | Sub 2 | resources | Segments describing human and material resources of national healthcare systems. | "And then you don't have staff in the hospitals because people have migrated already, especially nurses. Everyone from Macedonia, that is a nurse, has left the country permanently." | If the comment links the shortage of workers to CSO work 2.2.1.3 (shortages and changes (in human resources)) is to be used. |
| 5 | Main | determinants of POTMs´ access to healthcare | Segments describing factors facilitating or hindering the access to healthcare for people on the move. | "It is because of this huge problems, language, money, transportation problems." |  |
| 5.1 | Sub 1 | culture and language | Segments describing the influence of language and culture on the access to healthcare of people on the move including corresponding offers such as translators. | "It's very hard to provide mental health to someone who [doesn´t] speak your language. " | Overlapping with 2.1.3.3 (information and culture exchange) is possible, but in general, comments focusing on the role as mediator should be coded with 2.1.3.3, comments focusing on language and cultural problems should be coded with 5.1 |
| 5.2 | Sub 1 | information and integration | Segments describing the influence that integration of people on the move and their information/knowledge about the country, procedures and health have on their access to healthcare. | "There, it's like this. Identifying the children, ensuring their vaccination. And some of them didn't want to get vaccinated. There were misconceptions about vaccines." |  |
| 5.3 | Sub 1 | problems contacting POTM | Segments that describe the problem of contacting people on the move including their fears in order to provide them with healthcare. | "Sometimes you cannot even reach the people as you don't see them. If it happens to be, let's say, a big group of people being in a in a truck, for example. As a big group, they will isolate the group, they will not allow, for you to even reach to ask if anyone needs anything." | If the comment refers to people only transiting through the country and this is creating a problem 5.4 (change in location or legal status) is to be used |
| 5.4 | Sub 1 | change in location or legal status | Segments describing how changes in legal status or location/residence affects contact to people on the move and thus their access to healthcare. | "So, there is and then sometimes last, for example, last summer, here was much more people, that we have to organize the reception for them and then they leave after two days. So, you cannot do with them any activities. You don't do any kind of services, but you have to be here and provide them hygiene packages and organize accommodation and everything." |  |
| 5.5 | Sub 1 | provider | Segments describing the influence of health system workers regarding healthcare access for people on the move. | "But in practice, we have a lot of information, that they go to the doctor and then doctors said, no you have to go to health care, health insurance and make health card. Then we explain, no, they don't have." | Overlap with 1.1.3.2 (adherence to law and responsibilities) possible |
| 5.6 | Sub 1 | financial status | Segments describing the financial situation of people on the move and how this affects their access to healthcare. | "The problem is, access to drugs is problem. Because they have to pay co-payments also for the drugs and because of, for the chronic patients it is not easy to afford it." |  |
| 6 | Main | other actors | Segments describing the role and activities of other actors than civil society organizations and the healthcare system in healthcare support and related fields of work important for migration. | / |  |
| 6.1 | Sub 1 | intergovernmental organizations | Segments describing the role and activities of intergovernmental organizations in healthcare support and related fields of work important for migration. | "Although it's a state institution, the social workers, doctors and everyone else are paid by IOM over the state." | Because there is only one instance a comment on the European Union is coded with this category as well. |
| 6.2 | Sub 1 | academia | Segments describing the role and activities of academia in healthcare support and related fields of work important for migration. | "Data research gathering information and analysis, because data-based research are the most effective ones to. Especially in migration to be able to advocate for certain aspects. Like you are doing here for health." | Comments on kinder garden and schools are coded with this category as well. |
| 6.3 | Sub 1 | private sector | Segments describing the role and activities of the private sector in healthcare support and related fields of work important for migration. | "It is really a problem and because of that now, it is very new, I learned it from City1, I did not now it in City2, there are some private clinics, Syrian clinics. They are cheap and they are giving healthcare services to undocumented ones. Even for deliveries and some minor operations they are performing in that clinics. Some of the are legal, some of them are illegal." |  |
| 7 | Main | relationships | Segments that describe the (quality of) relationships and cooperation between civil society organizations and other civil society organizations and actors | "Also, we have a coordination for integration with ministries, with NGOs, with local level." |  |
| 7.1 | Sub 1 | public entities | Segments describing the relation and cooperation with public entities with the exception of the healthcare system. | "And we have connection with IOM concerning this medical, secondary level of medical healthcare and sometimes with additional food for all those, who stay in the transit centers." | Intergovernmental organizations are coded with this category as well. |
| 7.1.1 | Sub 2 | bad | Segments that describe the relationship, cooperation and communication of civil society organizations with governmental in a negative way. | "So, every time after we have an action plan with measure that how to improve integration, which also include the healthcare of migrants and those governmental office send all those conclusions or reports, what we do, for last years to the, to our government and they see that, but they don't do anything about that." |  |
| 7.1.1.1 | Sub 3 | obstruction of CSO work | Segments that describe how civil society organizations´ work is directly and purposefully obstructed by public entities. | "Regarding that part, that is what we are being seeing systematically is that, we are being systematically more and more barriers from the side of the government in order to ensure that the CSOs they face more barriers to access different state managed facilities." |  |
| 7.1.1.2 | Sub 3 | instrumentalization of CSO work | Segments that describe how civil society organizations´ work is instrumentalized by public entities for other purposes than health support for people on the move | "The government also the same time wants to hide things behind the wall. In a way they will probably, in order to show that they are providing service to people. I know there have been approaches from the government both to civil society organizations." |  |
| 7.1.2 | Sub 2 | good | Segments that describe the relationship, cooperation and communication in a good way such as good quality communication, facilitators for good cooperation or suggestions on how to improve cooperation and communication. | "But if you make, if you have a strong bond with them and if your organization is stronger, then probably it is being easier, to take those permission." |  |
| 7.2 | Sub 1 | with the healthcare system | Segments describing the relationship, cooperation and communication with the healthcare system. | "Therefore, we said that we could assist the health directorate in vaccination, and we planned a screening with them. Both screening and vaccination." |  |
| 7.3 | Sub 1 | with CSOs | Segments describing the relationship, cooperation and communication among civil society organizations. | "The NGOs, we are quite helpful, I would say, to each other. We are also giving joint statements." |  |
| 7.4 | Sub 1 | with POTM | Segments describing the relationship, cooperation and communication with people on the move. | "With the refugees and migrants, you know, we have a good communication because recognize the Organization1-logo like a very strong logo." |  |
| 7.5 | Sub 1 | with the general public | Segments describing the relationship, cooperation and communication with the domestic population or institutions of society such as the church. | "So, our citizens support this organization. It's quite well known locally. As we assist also local population in high social risk or living below the line of poverty." |  |
